# Exploring the Promotive Effects and Mechanisms of Different Polyphenolic Extracts from *Prinsepia utilis* Royle Seed Shell on Tyrosinase

**DOI:** 10.3390/foods11244015

**Published:** 2022-12-12

**Authors:** Shuang Ma, Xiuqing Zheng, Yuanyue Zhang, Shuai Zhao, Junjie Yi, Shengbao Cai

**Affiliations:** Faculty of Food Science and Engineering, Kunming University of Science and Technology, Kunming 650500, China

**Keywords:** *Prinsepia utilis* Royle, tyrosinase activity, molecular docking, molecular dynamics simulation, UHPLC-ESI-HRMS/MS

## Abstract

*Prinsepia utilis* Royle (*P. utilis*) is commonly used as a food ingredient and herbal medicine according to folk records, yet little research has been done on the seed shell, a processing waste. The aim of this study was to investigate the distribution of polyphenolic components and the tyrosinase activation activity of different extracts from the seed shell by UHPLC-ESI-HRMS/MS, in vitro tyrosinase activity assay, molecular docking and molecular dynamics. A total of 16 phytochemicals were identified, of which (+)-catechin and (−)-epicatechin were the major polyphenolic compounds. Both the esterified and insoluble bound polyphenols exhibited tyrosinase activation activity, and the esterified polyphenols showed better tyrosinase activation activity. (+)-Catechin and (−)-epicatechin might be the main activators of tyrosinase, both of which may act as substrate to affect tyrosinase activity. By molecular docking and molecular dynamics simulation studies, (+)-catechin and (−)-epicatechin can be efficiently and stably bound to the tyrosinase active site through hydrogen bonds, van der Waals forces and π-bonds. The results of this study may not only provide a scientific basis for exploring *P. utilis* seed shell as a potential activator of tyrosinase, but also contribute to the high value utilization of *P. utilis* processing by-products.

## 1. Introduction

Melanin, a polyphenolic biopolymer, is a dark brown pigment found in animal skin and hair that plays a key role in protecting the skin from DNA damage caused by ultraviolet light [1]. Insufficient melanin deposition can greatly reduce skin color and thus affect aesthetics and even cause a series of hypopigmented diseases, such as vitiligo, albinism, piebaldism, Hermansky-Pudlak syndrome, Griscelli syndrome, pityriasis alba and others [2]. Tyrosinase is a key enzyme in melanin biosynthesis and is also associated with a range of important natural reactions, such as fruit and vegetable browning, animal wound healing, insect development, human aging and human disease [3]. The process included in melanogenesis by this enzyme involves first catalyzing the production of o-dihydroxyphenylalanine (L-DOPA) from tyrosine, which is then further oxidized to dopaquinone and eventually melanin [4]. When local tyrosinase activity is affected, epidermal melanin deposition is hampered, thus affecting people’s healthy life. Currently, there are few clinical drugs that can effectively increase tyrosinase activity to promote melanin production. Statistically, psoralen is a widely used drug for the treatment of pigment deficiency in current clinical practice, however, it is usually accompanied by some adverse side effects [5]. Therefore, it makes sense to explore functional components from food materials to safely and effectively activate tyrosinase activity.

Polyphenols in natural products can usually be used as substrates for oxidative reactions with tyrosinase to produce quinones and eventually melanin [6]. This is also an important cause of enzymatic browning of foods [7]. Polyphenols are important secondary metabolites in plant growth, widely distributing in fruits, vegetables, and have been shown to possess a variety of biological activities [8]. This kind of natural product can be commonly divided into four groups, namely phenolic acids, flavonoids, stilbenes and lignans, and those compounds, as functional food dietary supplements, are found to possess either promotive or inhibitive effects on tyrosinase [9]. Some phenolic compounds, such as arbutin, ellagic acid and isorhamnetin, inhibit tyrosinase activity, while other phenolics, such as apigenin, lignans, kaempferol and quercetin, promote tyrosinase activity [10,11,12]. The ethyl acetate component isolated from the leaves of *Cespedesiaspathulata* (Ochnaceae) and its major polyphenolic components have been reported to promote tyrosinase activity well [13]. Van Staden et al. also found that asparagus alcoholic extracts and their phenolic components have a strong activating effect on tyrosinase [14]. However, it has also been reported that both the methanolic extract of *Flemingia philippinensis* roots and the polyphenolic components, chalcone derivatives, showed good inhibition of tyrosinase [15]. Fan et al. found that polyphenolic extracts from *Lonicera japonica* significantly inhibited tyrosinase activity [16]. According to results of these studies, it is clearly suggested that different plants and phenolic compositions may have different effects (promotion or inhibition) on tyrosinase, and plant extracts containing specific polyphenol components with promotive effects on tyrosinase may potentially act as safe activators to improve pigmentation disorders.

*Prinsepia utilis* Royle (*P. utilis*), a member of the *Rosaceae* family of shrubs, widely distributes at high altitudes in southwest China and parts of India [17], and is a good source of antioxidants [18]. In folk records, *P. utilis* is often used as a food ingredient or herbal medicine for treatment of rheumatism, pain, arthritis, bone disorders and joint disorders [19]. The leaves of *P. utilis* have been reported to have good antioxidant and anti-osteoporotic activities and phytochemical screening revealed that the leaves contain phytoconstituents such as flavonoids, tannins, terpenoids, β-sitosterol and ursolic acid, which might be a good source of phytopharmaceutical products [19]. Zhang et al. identified a variety of phenolic substances, such as rutin, quercetin-3-*O*-glucoside, isorhamnetin-3-*O*-rutinoside, cyanidin-3-*O*-glucoside and cyanidin-3-*O*-rutinoside, in the fruits with good antioxidant and hypoglycemic activities [20]. The seeds of *P. utilis* are the most valuable and useful part, which are often used to extract oil for daily consumption with a variety of benefits, such as antibacterial activity [21] and immunosuppression [22]. However, during the production of seed oil, a large amount of industrial waste is generated and discarded, especially the seed shell [23], which not only causes a waste of natural resources but also puts great pressure on the environment. Therefore, it is necessary to explore the potential usage of this food by-product. Zheng et al. have reported that the seed shell of *P. utilis* were rich in polyphenols, which have a good α-glycosidase inhibitory activity, and rutin was considered as its potential active substance [24]. However, it is not clear about the effect of the seed shell of *P. utilis* and the main components on tyrosinase activity and their underlying mechanism. Therefore, the aim of this research was to investigate the effects of different polyphenolic extracts from the seed shell of *P. utilis* on tyrosinase activity, to identify the main components through in silico screening, and to further determine the effects and mechanisms of the main components on tyrosinase through in vitro and molecular dynamics experiments. Results of this study may further expand the utilization and elevate the economic value of the seed shell from *P. utilis*, a type of food processing by-product.

## 2. Materials and Methods

### 2.1. Chemicals and Reagents

Formic acid, Folin-Ciocalteu reagent, methanol and acetonitrile were purchased from Merck (Darmstadt, Germany). Tyrosinase (from mushroom, EC1.14.18.1, ≥500 units/mg protein) was purchased from Shanghai Ruiyong Biotechnology Co., Ltd. (Shanghai, China). Levodopa (L-DOPA, purity ≥ 99.0%) was purchased from Beijing J&K Scientific Co., Ltd. (Beijing, China). All chemical standards, including (+)-catechin (purity ≥ 98.0%) and (−)-epicatechin (purity ≥ 98.0%), used in this study were purchased from Chengdu Bide Biotechnology Co., Ltd. (Chengdu, China). All other chemicals used in this study were of analytical grade.

### 2.2. Preparation of Sample

**Figure 1:**
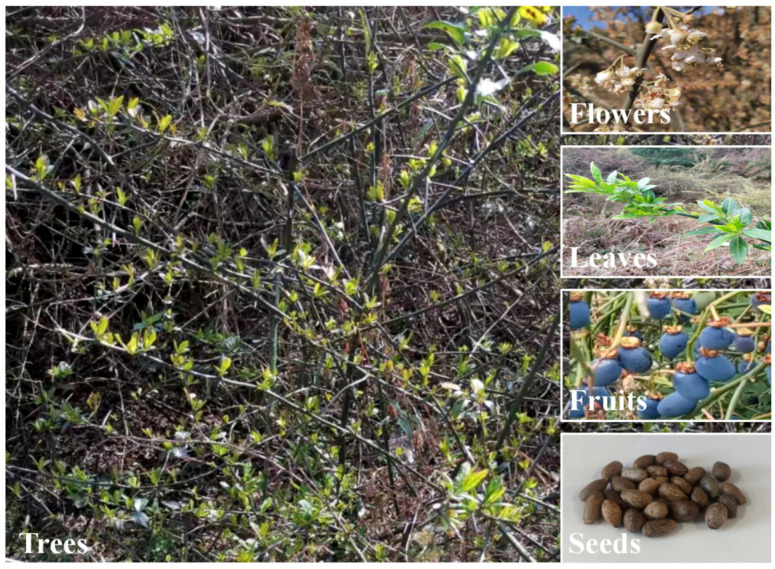
Picture with *Prinsepia utilis* Royle trees, flowers, leaves, fruits and seeds.

The seeds of *P. utilis* were obtained from Lijiang city, Yunnan, China, in May 2021. They were authenticated by Dr. Y.P. Liu, Kunming Institute of Botany, CAS. A specimen (No. kust20210504-16) was stored at the Faculty of Food Science and Engineering, Kunming University of Science and Technology. The apparent morphology of the various parts of *P. utilis* is shown in Figure 1, and the plant name was also checked online (http://www.theplantlist.org/tpl1.1/record/rjp-13504 (accessed on 11 May 2021). The seed shell obtained by manual peeling was powdered and passed through a 60-mesh sieve. Then, the powder was stored at −20 °C for subsequent experiments. The extraction steps of the free polyphenols (TF), the esterified polyphenols (TE) and the insoluble bound polyphenols (TI) were referred to the method of Zhang et al. [25].

### 2.3. Characterization and Quantification of Phytochemical Components with UHPLC-ESI-HRMS/MS

Three different states of polyphenols in *P. utilis* seed shell were identified by using a Thermo Fisher Ultimate 3000 UHPLC System coupled with Q-Exactive Orbitrap mass spectrometer (Thermofisher Scientific, Bremen, Germany). The polyphenolic compounds in the three samples were separated by a Poroshell 120 SB-C 18 column (2.1 × 100 mm, 2.7 μm, USA). The 0.5% formic acid water (phase A) and acetonitrile (phase B) were used as follows: 0–2 min, 5% B; 2–12 min, 5%–35% B; 12–13 min, 35%–5% B; 13–16 min, 5% B. The injection volume was 2.0 μL and the flow rate was 0.2 μL/min. Full MS scans were performed from *m/z* at 50 to 1000. The main parameter settings for MS were the same as in our previous report [26].

### 2.4. Measurement of Total Polyphenolic Contents and Total Flavonoid Contents

The total polyphenolic contents (TPCs) and the total flavonoid contents (TFCs) of three polyphenolic extracts were determined by the identical methods of Liu et al. [27]. TPCs and TFCs were expressed as mg gallic acid equivalent (GAE)/g of dry extract and mg rutin equivalent (RE)/g dry extract, respectively.

### 2.5. Measurement of Mushroom Tyrosinase Activity

The tyrosinase activity was determined by referring to the previously reported method with appropriate modifications [28]. The 40 μL of tyrosinase solution (100 U/mL) was mixed with 40 µL of *P. utilis* seed shell extract (50, 100, 150 and 200 µg/mL). The reaction mixture was completed by adding 40 µL of L-DOPA solution (3 mM) and adjusting the volume to 200 µL with phosphate buffer (0.2 M, pH 6.8). The reaction was carried out at 37 °C for 30 min and the absorbance values were recorded at 475 nm with a Spectra Max M5 microplate reader (Molecular Device, Sunnyvale, CA, USA). Blank group (system containing PBS, enzyme and L-DOPA solution), blank control group (system containing PBS and L-DOPA solution), and sample control group (system containing PBS, sample solution and L-DOPA solution) were also set up. The relative enzyme activity of each sample group was calculated using the following formula:

Relative enzyme activity (%) = [(B_1_ − B_2_)**/**(A_1_ − A_2_)] × 100

where A_1_ = absorbance of the blank group, A_2_ = absorbance of the blank control group, B_1_ = absorbance of the sample group and B_2_ = absorbance of the sample control group. The effective concentration of each sample to promote relative enzyme activity at 150% was calculated as EC_50_ value.

### 2.6. Enzyme Kinetic Analysis

The enzyme kinetics was determined by referring to the previously reported method with appropriate modifications [13]. The reactions were performed in 96-well plates with 40 μL of different concentrations of L-DOPA (1, 1.5 and 2 mmol/L), 40 μL of different concentrations of sample solution, 40 μL of mushroom tyrosinase solution (100 U/mL) and 80 μL of PBS buffer (pH = 6.8), respectively. As described in Section 2.5, a blank group, a blank control group and a sample control group were set up. The initial rate of dopachrome formation in the reaction mixture was determined as an increase in absorbance at a wavelength of 475 nm per minute by using a microplate reader. The Michaelis constant (K_m_) and maximal velocity (V_max_) of tyrosinase activity were determined by Lineweaver-Burk plots using different concentrations of (+)-catechin and (−)-epicatechin as substrates.

### 2.7. Molecular Docking

Molecular docking was performed using AutoDock 4.2 software (San Diego, CA, USA) [29]. The 3D structure of tyrosinase (PDB: 2Y9X) was downloaded from the RCSB database (http://www.rcsb.org/pdb/home/home.do (accessed on 7 October 2022). Then, the existing ligands and water molecules in the downloaded 3D structure of tyrosinase were removed, thereby obtaining a pure 3D structure of tyrosinase for molecular docking. The 3D structures of L-DOPA (PubChem ID: 6047), (+)-catechin (PubChem ID: 9064) and (−)-epicatechin (PubChem ID: 72276) were obtained from the PubChem database (https://www.ncbi.nlm.nih.gov/ (accessed on 7 October 2022). The ligands and tyrosinase were added to the hydrogen atoms and Gastieger charges and optimized by using the GAFF force field [30]. Subsequently, the entire tyrosinase protein was applied as a potential binding site using a blind docking method. The grid coordinates used for molecular docking were x = −10.004, y = −28.28, z = −43.443 with dimensions of 40 × 40 × 40 Å. The conformations and interactions obtained were visualized using PyMOL (version 2.3.1) (https://PyMOL.org/ (accessed on 9 October 2022).

### 2.8. Molecular Dynamics (MD) Simulation

To validate the results of molecular docking and to reveal the mechanism of tyrosinase activation by (+)-catechin and (−)-epicatechin, molecular dynamics simulations were performed for 50 ns. MD simulations were performed according to the method of Zhou et al. with some minor modifications [31]. The system trajectories were analyzed using the GROMACS 19.5 package [32]. The protein/complex was placed in a rectangular box of TIP3P water molecules with a minimum distance of 1.5 Å between any solute atom and the edge of the periodic box. Counter ions were added to neutralize the total charge of the system, and then the steepest descent method was used to minimize the energy to eliminate undesirable contacts and spatial conflicts with a maximum energy of 1000.0 kJ/mol/nm. The system was equilibrated by two steps: (1) regular system synthesis (NVT, 0.2 ns) and (2) isothermal-isobaric (NPT, 1 ns). Constant temperature and pressure (310.15 K, 1 bar) were achieved in the complex system. V-rescale and Parrinello-rahman barometer were used to control the temperature and pressure in the complex system [33]. MD simulations were then performed. Data analyses included root mean square deviation (RMSD), root mean square fluctuation (RMSF), radius of rotation (Rg) and surface solvent accessible area (SASA).

### 2.9. Statistical Analysis

Each experiment was repeated three times, and data were expressed as mean ± standard deviation (SD). All data were subjected to one-way ANOVA by using Origin 8.5 software (Origin Lab, Northampton, MA, USA), and Tukey’s test was used to determine significant differences (*p* < 0.05).

## 3. Results and Discussion

### 3.1. Identification and Quantification of Phenolic Compounds and TFCs and TPCs

**Figure 2:**
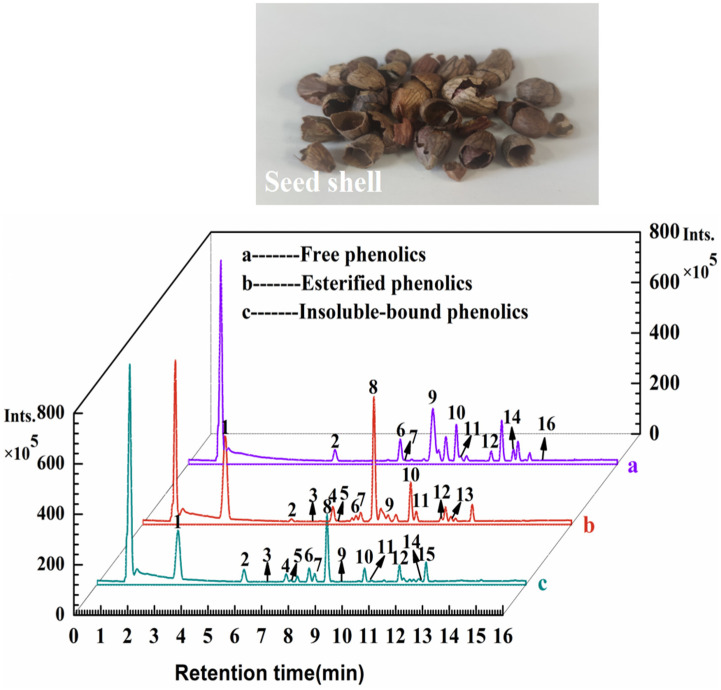
Basal peak chromatograms of the TF (**a**), TE (**b**), and TI (**c**) extracts from the seed shell of *Prinsepia utilis* Royle.

**Figure 3:**
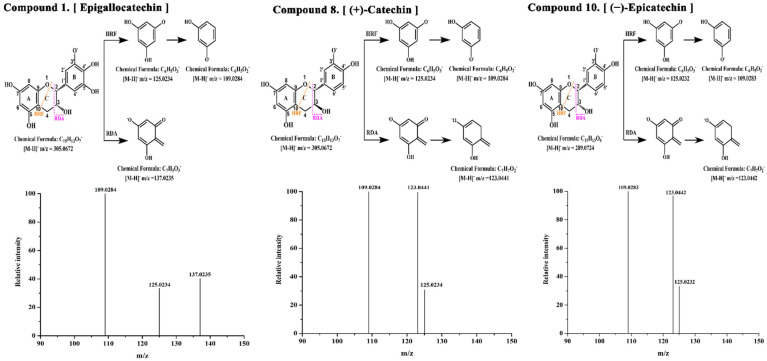
The secondary mass spectra of main polyphenolic compounds epigallocatechin, (+)-catechin and (−)-epicatechin, respectively, in negative ion mode.

The chemical composition of TF, TE and TI in the seed shell of *P. utilis* was identified by UHPLC-ESI-HRMS/MS. The base peak chromatograms are shown in Figure 2, and the *m/z*, retention times and MS/MS fragments are summarized in Table 1. A total of 16 compounds were identified by reference to commercial standards, massbank (https://massbank.eu/MassBank/Search) or previous reports, including three phenolic acids and their derivatives (compounds **2**, **5**, **6**), eleven flavonoids (compounds **1**, **3**–**4**, **7**–**10**, **12**, **14**–**16**), one aldehyde (compound **11**) and one phenolic metabolite (compound **13**). Among them, compound **6**, compound **9** and compound **10** had relatively higher peak areas in TF, indicating that these three compounds might be the main compounds in TF. Compound **6** ([M − H]^–^ *m/z* = 137.0235) was identified as salicylic acid with characteristic fragment *m/z* of 93.0334 and 65.0384, respectively [34]. Compound **9** ([M − H]^−^ *m/z* = 577.1368) is procyanidin B1 isomer I with characteristic fragment ions of 125.0234, 289.0722 and 407.0779, respectively [35]. Compound **10** ([M − H]^−^ *m/z* = 577.1368) is (−)-epicatechin with characteristic fragment *m/z* of 109.0283, 123.0442 and 125.0232, respectively. Compound **1**, compound **8** and compound **10** ((−)-epicatechin) had relatively higher peak areas in TE and TI, indicating that these three phenolic compounds may be the major compounds in TE and TI extracts. Compound **1** ([M − H]^−^ *m/z* = 305.0672) and compound **8** ([M − H]^–^ *m/z* = 289.0723) were identified as epigallocatechin and (+)-catechin, respectively. Among them, the characteristic fragments *m/z* of epigallocatechin were 109.0284, 137.0235 and 125.0234, and (+)-catechin were 109.0284, 123.0441 and 125.0234, respectively. The cleavage patterns of epigallocatechin, (+)-catechin and (−)-epicatechin are shown in Figure 3. Epigallocatechin, on the one hand, underwent heterocyclic ring fission (HRF), and the 1,4 bond of the C ring was broken, shedding a 5-(2-Hydroxypropyl) benzene-1,2,3-triol and thus obtaining the characteristic ionic fragment *m/z* of 125.0234, on the basis of which it is de-hydroxylated to obtain a characteristic ionic fragment *m/z* of 109.0284 [36]. On the other hand, by Retro-Diels-Alder (RDA) cleavage, the 1,3 bond of the C ring was broken and a 3,4,5-trihydroxyphenethyl alcohol was shed to obtain the characteristic ion fragment 137.0235. Similarly, (+)-catechin or (−)-epicatechin was cleaved by heterocyclic ring fission (HRF), and a 4-(2-hydroxypropyl) benzene-1,2-diol was shed to obtain the characteristic ionic fragment *m/z* of 125.0234 or 125.0232, on the basis of which it is de-hydroxylated to obtain the characteristic fragment *m/z* of 109.0284 or 109.0283 [36]. The characteristic ionic fragment of 109.0284 or 109.0283 was obtained by Retro-Diels-Alder (RDA) fission to shed 4-(2-hydroxyethyl) benzene-1,2-diol and on this basis another oxygen atom was shed to obtain the characteristic ion fragment *m/z* of 123.0441 or 123.0442. Compound **2** ([M − H]^–^ *m/z* = 153.0185) was identified as protocatechuic acid with characteristic fragments *m/z* of 108.0205, and 109.0289; compound **3** ([M − H]^−^ *m/z* = 285.0409) was identified as luteolin with characteristic fragments *m/z* of 132.0208, 175.0394 and 199.0395 [35]. Compound **7** ([M − H]^–^ *m/z* = 577.1366) was identified as procyanidin B1 with characteristic fragments *m/z* of 125.0234, 289.0724 and 407.0777 [35].

The contents of the target polyphenolic compounds in the TF, TE and TI were quantified using the standard curves of the corresponding standard. The quantitative results are shown in Table 1. Epigallocatechin, (+)-catechin and (−)-epicatechin were present in *P. utilis* seed shell at high contents, and it can be judged that they were the three main polyphenolic compounds in the seed shell of *P. utilis*. Among them, epigallocatechin was detected in TE and TI, and (+)-catechin and (−)-epicatechin were detected in all three polyphenolic extracts. The contents of epigallocatechin in TE and TI extracts were 6472.58 ± 173.40 and 4013.03 ± 102.94 μg/g, respectively, accounting for 35.47% and 33.77% of the total polyphenolic compounds. The contents of (+)-catechin in TF, TE and TI extracts were 123.90 ± 1.27, 6413.34 ± 193.50 and 3620.13 ± 40.92 μg/g, respectively, accounting for 1.37%, 35.15% and 30.46% of the total phenolic compounds in the extracts. The content of (−)-epicatechin was 1915.71 ± 28.15, 2088.99 ± 33.21 and 822.69 ± 9.87 μg/g in TF, TE and TI extracts, accounting for 21.18%, 11.45% and 6.92% of the total phenolic compounds in the extracts, respectively. This is consistent with the results of a previous study where there were differences in the contents of common main polyphenolics obtained from the same plant material with different extraction methods [37]. Epigallocatechin was not detected in TF, while it was detected at higher levels in both TE and TI groups, which may be because the epigallocatechin is bound to plant cell walls or other biomolecules, which can be extracted by hydrolysis with strong bases [38]. According to the above results, (+)-catechin and (−)-epicatechin were the main polyphenolic compounds in the shell, which have been reported to have various biological activities, such as antioxidant [39], anti-inflammatory [40,41] and hypoglycemic activities [42]. Moreover, previous studies also found that both catechin and epicatechin possessed a good activating effect on tyrosinase [13,14].

Table 1 summarizes the TPC and TFC of each fraction. TPC was expressed as mg gallic acid equivalent (GAE)/g dry weight, and TFC was expressed as mg rutin equivalent (RE)/g dry weight. Among the three polyphenol extracts, the TPC of TE was significantly higher than that of TF and TI (*p* < 0.05), about 351.45 ± 14.91 mg GAE/g, which was 0.72 and 0.64 times higher than the TPC contents of TF and TI, respectively. The TFC of TF and TE were similar, 200.86 ± 10.25 mg RE/g and 205.34 ± 11.26 mg RE/g, respectively, with no significant difference (*p* < 0.05). However, the TFC of TI was significantly lower than that of TF and TE (*p* < 0.05). Those results indicated that TE was the main form of polyphenols present in the seed shell of *P. utilis*, which was consistent with previous studies in which the major phenols in tea seeds and sesame bark were esterified phenols [35,43]. However, free phenols have also been reported to be present in the major form in raspberry pomace plants and insoluble bound phenols in oil palm fruits [26,44]. The discrepancies between these reports and the current study may be due to different plant species or different growth environments [45,46]. In summary, it can be concluded that the seed shell of *P. utilis* was rich in polyphenolic compounds, which may be a good source of dietary phenolics.

**Table 1:** The identified chemical constituents, total polyphenols and total flavonoids in the TF, TE and TI extracts from the seed shell of *Prinsepia utilis* Royle.

Peak No.	Compounds	TR * (min)	Error (ppm)	Molecular Formula	[M − H]^–^ (*m/z*)	MS/MS Fragment Ions	Reference	Extracts		
								TF (μg/g)	TE (μg/g)	TI (μg/g)
1	Epigallocatechin	3.08	5.215	C_15_H_14_O_7_	305.0672	109.0284, 137.0235, 125.0234	Standard	Trace	6472.58 ± 173.40 ^b^	4013.03 ± 102.94 ^a^
2	Protocatechuic acid	5.56	1.862	C_7_H_6_O_4_	153.0185	108.0205, 109.0289	Standard	872.89 ± 5.52 ^a^	881.08 ± 7.32 ^a^	948.45 ± 12.22 ^b^
3	Luteolin	6.40	5.808	C_15_H_10_O_6_	285.0409	132.0208, 175.0394, 199.0395	[35]	Trace	332.14 ± 7.08 ^a^	707.83 ± 12.36 ^b^
4	Dihydrokaempferol	7.10	1.715	C_15_H_12_O_6_	287.0567	201.0553, 177.0552, 125.0235	[43]	Trace	314.28 ± 4.43 ^a^	203.35 ± 9.11 ^b^
5	DL-3-(4-Hydroxyphenyl)lactic acid	7.28	3.174	C_9_H_10_O_4_	181.0501	134.0364, 72.9918, 135.0436	[47]	Trace	157.24 ± 2.32 ^a^	194.98 ± 7.22 ^b^
6	Salicylic acid	7.97	1.529	C_7_H_6_O_3_	137.0235	93.0334, 65.0384	[34]	1361.93 ± 11.81 ^c^	490.48 ± 5.48 ^a^	900.95 ± 17.52 ^b^
7	Procyanidin B1	8.13	4.414	C_30_H_26_O_12_	577.1366	125.0234, 289.0724, 407.0777	[35]	255.44 ± 2.96 ^b^	528.31 ± 1.62 ^c^	115.35 ± 1.04 ^a^
8	(+)-Catechin	8.63	5.657	C_15_H_14_O_6_	289.0723	109.0284, 123.0441, 125.0234	Standard	123.90 ± 1.27 ^a^	6413.34 ± 193.50 ^c^	3620.13 ± 40.92 ^b^
9	Procyanidin B1 isomer I	9.16	4.726	C_30_H_26_O_12_	577.1368	125.0234, 289.0722, 407.0779	[35]	3478.77 ± 14.51 ^c^	26.26 ± 2.62 ^b^	4.17 ± 0.04 ^a^
10	(−)-Epicatechin	10.00	5.865	C_15_H_14_O_6_	289.0724	109.0283, 123.0442, 125.0232	Standard	1915.71 ± 28.15 ^b^	2088.99 ± 33.21 ^c^	822.69 ± 9.87 ^a^
11	4-Hydroxybenzaldehyde	10.14	0.199	C_7_H_6_O_2_	121.0284	108.0205, 93.0331, 121.0286	[48]	163.34 ± 4.59 ^a^	55.52 ± 0.83 ^b^	99.80 ± 1.36 ^c^
12	Dihydroquercetin	11.16	5.414	C_15_H_12_O_7_	303.0516	125.0234, 150.0316	Standard	46.80 ± 1.13 ^a^	128.03 ± 4.08 ^b^	146.96 ± 5.63 ^c^
13	Urolithin C	11.58	5.185	C_13_H_8_O_5_	243.0301	127.0544, 199.0397	Standard	Trace	359.04 ± 9.37	Trace
14	Quercetin-3-*O*-hexoside	12.12	4.400	C_21_H_20_O_12_	463.0891	300.0279, 301.0338, 271.0251	[49]	760.22 ± 11.76 ^b^	Trace	55.02 ± 1.74 ^a^
15	Luteoloside	12.23	4.456	C_21_H_20_O_11_	447.0942	285.0406, 284.0331	Standard	Trace	Trace	53.95 ± 1.16
16	Isorhamnetin-3-*O*-glucoside	13.24	4.040	C_22_H_22_O_12_	477.1045	314.0438, 271.0253, 315.0485	[50]	65.97 ± 1.34	Trace	Trace
Total Polyphenolic Content (mg GAE/g)								252.92 ± 13.71 ^a^	351.45 ± 14.91 ^b^	225.58 ± 9.99 ^a^
Total Flavonoid Content (mg RE/g)								200.86 ± 10.25 ^b^	205.34 ± 11.26 ^b^	159.57 ± 5.48 ^a^

* TR: retention time; TF, TE and TI denote free polyphenol, esterified polyphenol and insoluble-bound polyphenol forms, respectively. TPC: total polyphenolics content; TFC: total flavonoids content. All values are mean ± SD (n = 3), and values with different superscript letters (^a, b, c^) in each row indicated significant differences between samples (*p* < 0.05). Compound **1** was semi-quantified using gallic acid standard. Compound **2** was quantified using protocatechuic acid standard. Compounds **3** and **15** were quantified and semi-quantified using luteolin standard. Compound **4** was semi-quantified using dihydromyricetin standard. Compound **6** was quantified using salicylic acid standard. Compounds **7**, **8** and **9** were quantified and semi-quantified by (+)-catechin standard. Compound **10** was quantified by (−)-epicatechin standard. Compounds **5** and **11** were quantified semi-quantitatively using p-hydroxybenzoic acid standard. Compounds **12** and **14** were semi-quantified by quercetin standard. Compound **13** was semi-quantified by ellagic acid standard. Compound **16** was semi-quantified by isorhamnetin standard.

### 3.2. Tyrosinase Activity Determination

**Figure 4:**
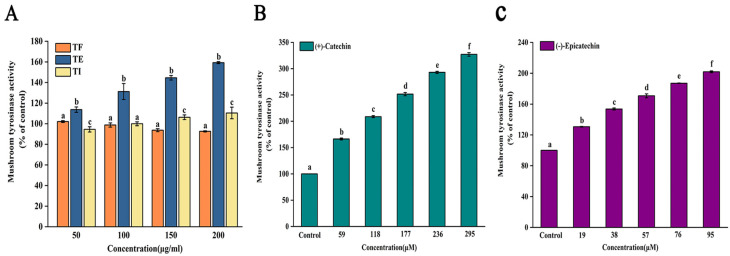
Activities effect of three polyphenolic extracts (**A**) and main components ((+)-catechin (**B**) and (−)-epicatechin (**C**)) from *Prinsepia utilis* Royle seed shell toward tyrosinase. Values are shown as the mean ± SD (n = 3). TF, the free polyphenols; TE, the esterified polyphenols; TI, the insoluble bound polyphenols. The different letters indicated significant differences (*p* < 0.05).

Figure 4A shows the effect of different forms of phenolics from the seed shell of *P. utilis* on tyrosinase activity. Below the concentration of 200 μg/mL, TE and TI showed activation effect on tyrosinase and both were dose dependent, while TF did not exhibit significant activation effect on tyrosinase activity (*p* < 0.05). Among TE and TI, the activation effect of TE on tyrosinase (EC50 value of 187.89 ± 7.87 μg/mL) was better, with a relative activation rate of 159.24 ± 0.99% at a concentration of 200 μg/mL, whereas the relative activation rate of TI was 110.45 ± 5.59%, indicating that the activation effect of TE on tyrosinase was significantly better than that of TI (*p* < 0.05). In order to further reveal the main components of TE and TI that took responsibility for activation effects, their main compounds were screened. The results are shown in Figure 4B,C, (+)-catechin and (−)-epicatechin showed significant activation effects on tyrosinase with EC50 values of 32.50 ± 1.70 μM and 36.71 ± 0.43 μM, respectively (*p* < 0.05). The results of this experiment were similar to those reported in a previous study [14]. The alcoholic extract of Aspalathus linearis showed a good activation effect on tyrosinase with EC50 values of 140.60 ± 2.69 μg/mL, which was slightly better than the TE of the seed shell from *P. utilis* in this experiment; however, the main compounds (+)-catechin and (−)-epicatechin of *P. utilis* seed shell were significantly better than the main component of *Aspalathus linearis*, Aspalathin (EC50 of 264.75 ± 4.56 μM) [14]. Moreover, it is reported that the ethyl acetate fraction of *Cespedesia spathulata* leaves, rich in ±catechin, activated the tyrosinase activity about 50% at 320.19 μg/mL [13]. Due to the differences in plant species and extraction methods, the activation effect on tyrosinase may be different, which eventually led to the discrepancy between the present experimental results and the reported results. In conclusion, the seed shell of *P. utilis* and its main components (+)-catechin and (−)-epicatechin can effectively activate tyrosinase and can be further developed and studied as tyrosinase activators.

### 3.3. Enzyme Kinetic Analysis

**Figure 5:**
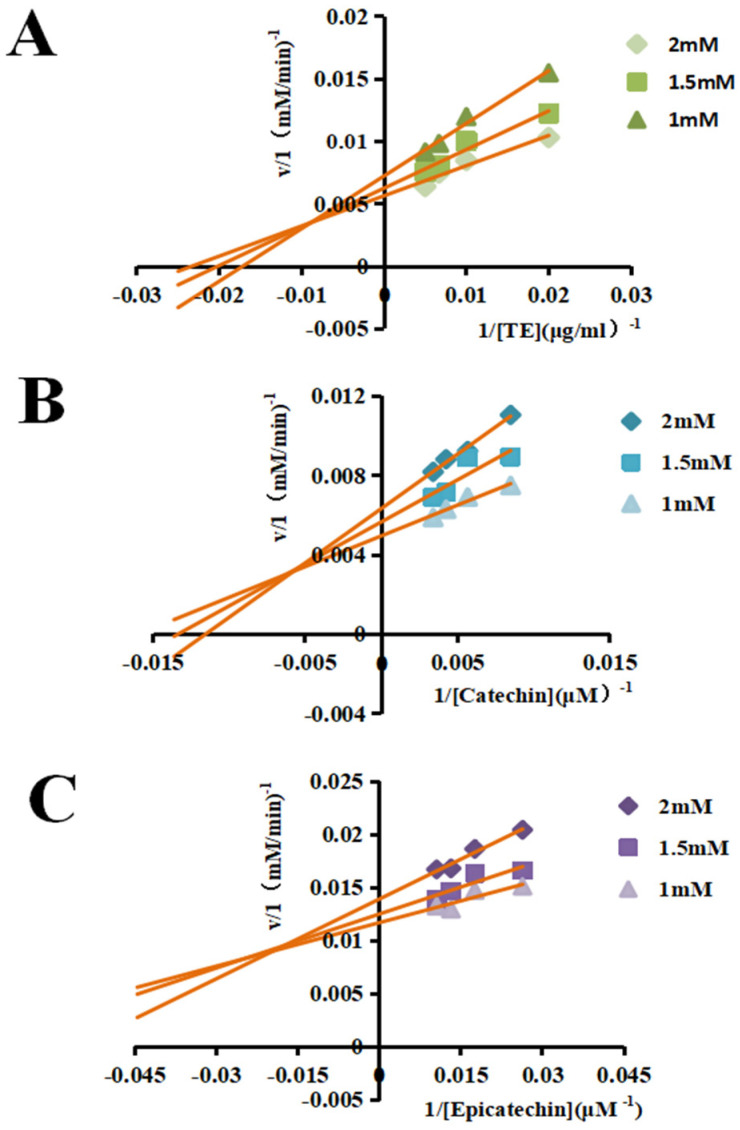
Experiments similar to the Lineweaver-Burk method were used to evaluate the activity of the samples against tyrosinase. The substrate consisted of TE (**A**), (+)-Catechin (**B**), (−)-Epicatechin (**C**), and concentrations of L-DOPA for each plot were 1, 1.5 and 2 Mm.

According to the above results, the TE and its main components, (+)-catechin and (−)-epicatechin, could significantly activate the tyrosinase activity. Therefore, enzyme kinetic analysis was performed to further analyze the enzymatic reaction relationship and catalytic characteristics (the affinity binding force and catalytic rate) between tyrosinase, substrate and samples. It can be seen from Figure 5A and Table 2 that Km showed a decreasing trend and Vm showed an increasing trend as the concentration of L-DOPA increased, indicating that the binding affinity force and the catalytic rate of TE to tyrosinase increased, and the latter may be affected by the former. For (+)-catechin or (−)-epicatechin, as shown in Figure 5B,C and Table 2, the Km value decreased with decreasing L-DOPA concentration, indicating that (+)-catechin or (−)-epicatechin increased the binding affinity to tyrosinase as reported in the previous study [51], which may be because all (+)-catechin, (−)-epicatechin and L-DOPA can act as substrates for tyrosinase [13], and (+)-catechin or (−)-epicatechin compete with L-DOPA for the substrate binding site of tyrosinase. Meanwhile, (+)-catechin or (−)-epicatechin showed an increasing trend in the catalytic rate, consistent with the above result of TE, which further indicated that the binding affinity affects the catalytic rate of tyrosinase. However, there are some differences between TE and pure (+)-catechin or (−)-epicatechin in terms of changes in the reaction with tyrosine kinetics, and this phenomenon may be due to the fact that some other substances contained in TE (including substances other than (+)-catechin and (−)-epicatechin identified in this work and substances not identified) could affect the conformation or catalysis of tyrosinase, thus affecting the binding of the substrate to the enzyme and the catalytic characteristics, which needs further studies to comprehensively investigate. Overall, the results of this experiment further confirmed that both (+)-catechin or (−)-epicatechin and L-DOPA can be used as substrates for tyrosinase, and in the presence of both L-DOPA and (+)-catechin or (−)-epicatechin, the tyrosinase activity was affected due to the samples competing with L-DOPA for the substrate binding site of tyrosinase and thereby affecting the catalytic rate. Although there may be some influence by the other components in the TE, the above results are sufficient to confirm that (+)-catechin and (−)-epicatechin may make great contributions to the activation of tyrosinase by the TE from the *P. utilis* seed shell.

### 3.4. Molecular Docking

**Figure 6:**
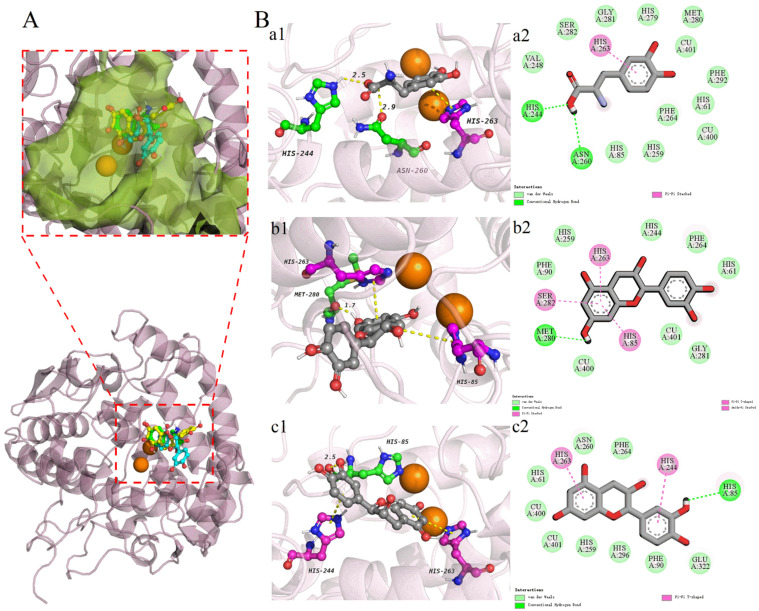
Molecular docking results of L-DOPA, (+)-catechin and (−)-epicatechin with tyrosinase active proteins. (**A**) is the pocket position of the three compounds bound to tyrosinase. Three-dimensional conformation diagrams of L-DOPA (**Ba1**), (+)-catechin (**Bb1**) and (−)-epicatechin (**Bc1**) forming different forces with tyrosinase. (**Ba2**,**Bb2**,**Bc2**) are two-dimensional conformation diagrams of hydrogen bonds, van der Waals forces and π-bonds formed by the three compounds with tyrosinase, respectively.

Results of the interaction of (+)-catechin, (−)-epicatechin and L-DOPA with tyrosinase are shown in Table 3 and Figure 6. The higher absolute values of affinity indicated stronger binding effect of the ligand to the active protein [52]. The affinities of L-DOPA, (+)-catechin and (−)-epicatechin with tyrosinase were −6.3 kcal/mol, −6.9 kcal/mol and −6.7 kcal/mol, respectively. This showed that the both affinities of (+)-catechin and (−)-epicatechin towards tyrosinase were higher than that of L-DOPA which is a substrate specifically recognized by tyrosinase [53], so it is concluded that both (+)-catechin and (−)-epicatechin bind well to tyrosinase, and (+)-catechin may have the best potential activation effect on tyrosinase.

Li et al. found that (+)-catechin and (−)-epicatechin as activators can bind to tyrosinase through hydrogen bonding, π-bonding and van der Waals forces, and the π-bonds were the key force for substrate recognition by tyrosinase [54]. Figure 6 demonstrates that L-DOPA, (+)-catechin and (−)-epicatechin form 1, 3 and 2 π-bonds with tyrosinase, respectively (Table 3). It can be seen that both (+)-catechin and (−)-epicatechin formed more π-bonds with the amino acid residues near the active site of tyrosinase than the substrate L-DOPA, and the (+)-catechin formed most π-bonds with the amino acid residues near the active site of tyrosinase. Thus, both (+)-catechin and (−)-epicatechin can catalyze tyrosinase activity as potential substrates, and (+)-catechin will catalyze tyrosinase better. Moreover, Ji et al. found that hydrogen bonds and van der Waals forces were the main forces formed by tyrosinase with ligands [55], and in this study, L-DOPA, (+)-catechin and (−)-epicatechin formed 2, 1 and 1 hydrogen bonds and 12, 8 and 9 van der Waals forces with tyrosinase, respectively. Buitrago et al. found that copper ion is the main active site of tyrosinase [56], and it can be seen from Table 3 that CU400, CU401 near the active site of tyrosinase copper ion formed van der Waals forces with L-DOPA, (+)-catechin and (−)-epicatechin. Thus, by intermolecular interaction force analysis, L-DOPA, (+)-catechin and (−)-epicatechin all formed hydrogen bonds, π-bonds and van der Waals forces with tyrosinase, and (+)-catechin may bind more strongly to tyrosinase. Taken together, it indicated that (+)-catechin and (−)-epicatechin as substrates can effectively activate tyrosinase activity and may be potential tyrosinase activators that deserve further study.

### 3.5. Molecular Dynamics

**Figure 7:**
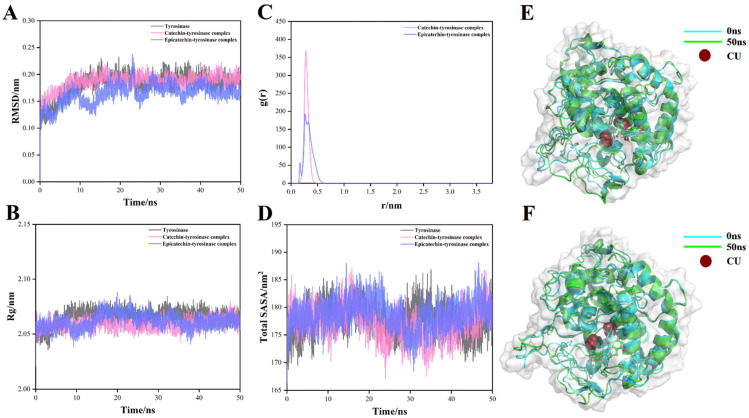
Molecular dynamics (50 ns) results of tyrosinase with (+)-catechin and (−)-epicatechin. (**A**) Root mean square deviation (RMSD, nm), (**B**) Radius of rotation (Rg), (**C**) Radial distribution function (RDF), and (**D**) Solvent accessible surface area (SASA) values. The molecular dynamics results of (+)-catechin-tyrosinase and (−)-epicatechin-tyrosinase system at 0ns and 50ns, respectively (**E**,**F**).

Based on molecular docking, molecular dynamics simulations were used to further investigate the conformational changes during the binding of the major compounds to the active protein. The RMSD method was used to determine the average deviation of the ligand-enzyme receptor conformation within 50 ns thus assessing the steady state of the ligand-receptor system [57]. Figure 7A shows that the RMSD values of the (+)-catechin-tyrosinase system and (−)-epicatechin-tyrosinase system exhibited an increasing trend at first, then decreased and finally reached the basic equilibrium during 50 ns, indicating that the ligand is bound to the active site of tyrosinase and the whole system was stable. Each class of protein has its own specific radius of gyration, which determines the tightness of the protein structure [58]. Throughout the simulation, the Rg values of the (+)-catechin-tyrosinase system and the (−)-epicatechin-tyrosinase system fluctuated without significant changes (Figure 7B), indicating that the ligand entering into the internal structure of the tyrosinase did not destroy its original structure, which is consistent with the observations in Figure 7E,F that the active pocket of copper ions did not change significantly and the binding of the study object to the tyrosinase remained stable. To further understand the binding stability of these two different compounds with tyrosinase, the distance between the interaction forces of (+)-catechin and (−)-epicatechin with tyrosinase were predicted. The results are shown in Figure 7C, the interaction distances of (+)-catechin and (−)-epicatechin with tyrosinase during the 50 ns MD simulation are around 0.28 and 0.26 nm, respectively, which indicated that the interaction force distance between the ligand and the active site was short, which, in turn, indicated a stronger binding between them. The exposure of amino acid residues to the solvent was evaluated by SASA (Figure 7D) [59]. It can be seen that the internal fluctuations of SASA for (+)-catechin-tyrosinase and (−)-epicatechin-tyrosinase system showed a trend of decreasing and then increasing fluctuations, which in contrast to the internal fluctuations of the tyrosinase system alone. The possible reason for this trend may be the gradual stabilization of the binding of the ligand to the protein with the increase of hydrophobicity and the discharge of solvent water. This indicated that the structure of the enzyme was not destroyed during the action and also illustrated the accuracy of the RG results in Figure 7C. The above results indicated that (+)-catechin and (−)-epicatechin did not change the protein conformation during binding to tyrosinase and were able to bind stably to exert their activating effects.

## 4. Conclusions

In this study, a total of 16 compounds were detected, among which (+)-catechin and (−)-epicatechin were the major polyphenolic compounds. Among the three polyphenol extracts of *P. utilis* seed shell, both the TE and the TI showed activation effects on tyrosinase, and the former showed a better activity. The (+)-catechin and (−)-epicatechin, as the main activators of tyrosinase, could act as substrates to compete with L-DOPA, thereby affecting the tyrosinase activity. Molecular docking and molecular dynamics results showed that (+)-catechin and (−)-epicatechin stably and effectively bound to the tyrosinase active site through three forces (hydrogen bonds, van der Waals force and π-bonds). In conclusion, the polyphenolic extracts, especially the TE, could be used as potential activators for the treatment of vitiligo, albinism, and other disorders related to pigmentation disorders.

## Figures and Tables

**Table 2:** Michaelis constant (Km) and maximum velocity (Vm) of tyrosinase activity in the presence of TE, (+)-catechin and (−)-epicatechin.

DOPA (in mM)	Km			Vm		
	TE	(+)-Catechin	(−)-Epicatechin	TE	(+)-Catechin	(−)-Epicatechin
1	58.40	62.02	11.17	138.89	200.00	85.47
1.5	49.05	74.32	11.68	158.73	175.44	80.00
2	42.32	87.05	18.02	175.44	158.73	71.94

**Table 3:** Binding affinity and binding sites of L-DOPA, (+)-catechin and (−)-epicatechin to tyrosinase.

	L-DOPA	(+)-Catechin	(−)-Epicatechin
Pubchem ID	6047	9064	72276
Affinity energy (kcal/mol)	−6.3	−6.9	−6.7
Number of hydrogen bonds	2	1	1
Amino acid residues involved in hydrogen bonding	HIS 244, ASN 260	MET 280	HIS 85
Number of hydrophobic interactions	12	8	9
Amino acid residues involved in hydrophobic interactions	HIS 85, HIS 259, PHE 264, CU 400, HIS 61, PHE 292, CU 401, MET 280, HIS 279, GLY 281, SER 282, VAL 248	CU 401, GLY 281, PHE 90, PHE 264, HIS 244, HIS 259, HIS 61, CU 400	CU 400, CU 401, HIS 259, HIS 296, PHE 90, GLU 322, PHE 264, ASN 260, HIS 61
Number of π-bonds	1	3	2
Amino acid residues involved in π-bonds	HIS 263	HIS 263, HIS 85, SER 282	HIS 244, HIS 263

## Data Availability

The data that support the findings of this study are available from the corresponding author upon reasonable request.
